# Long Noncoding RNA AFAP1-AS1 Is a Critical Regulator of Nasopharyngeal Carcinoma Tumorigenicity

**DOI:** 10.3389/fonc.2020.601055

**Published:** 2020-11-23

**Authors:** Min Fang, Minjun Zhang, Yiqing Wang, Fangqiang Wei, Jianhui Wu, Xiaozhou Mou, Yigan Zhang, Xiaodong Liang, Jianming Tang

**Affiliations:** ^1^ Department of Radiation Oncology, Zhejiang Provincial People’s Hospital, People’s Hospital of Hangzhou Medical College, Hangzhou, China; ^2^ Graduate Department, Bengbu Medical College, Bengbu, China; ^3^ The Reproductive Medicine Special Hospital of the First Hospital of Lanzhou University, Key Laboratory for Reproductive Medicine and Embryo, Lanzhou, China; ^4^ Department of Hepatobiliary and Pancreatic Surgery, Zhejiang Provincial People’s Hospital, People’s Hospital of Hangzhou Medical College, Hangzhou, China; ^5^ Department of the Otolaryngology, Zhongshan City People’s Hospital, Zhongshan Affiliated Hospital of Sun Yan-sen University, Zhongshan, China; ^6^ Key Laboratory of Tumor Molecular Diagnosis and Individualized Medicine of Zhejiang Province, Zhejiang Provincial People’s Hospital, People’s Hospital of Hangzhou Medical College, Hangzhou, China; ^7^ Clinical Research Institute, Zhejiang Provincial People’s Hospital, People’s Hospital of Hangzhou Medical College, Hangzhou, China; ^8^ The First School of Clinical Medicine, Lanzhou University, Lanzhou, China

**Keywords:** lncRNA AFAP1-AS1, KAT2B, YAP, nasopharyngeal carcinoma, nasopharyngeal carcinoma tumorigenicity

## Abstract

**Background:**

The long noncoding RNA actin filament associated protein 1 antisense RNA1 (AFAP1-AS1) is a critical player in various cancers. However, the clinical value and functional mechanisms of AFAP1-AS1 during the tumorigenicity of nasopharyngeal carcinoma (NPC) remain unclear. Here, we investigated the clinical application and potential molecular mechanisms of AFAP1-AS1 in NPC tumorigenesis and progression.

**Methods:**

The expression level of AFAP1-AS1 was determined by qRT-PCR in 10 paired fresh human NPC tissues and adjacent normal tissues. RNAscope was performed on 100 paired paraffin-embedded NPC and adjacent nontumor specimens. The biological functions of AFAP1-AS1 were assessed by *in vitro* and *in vivo* functional experiments. RNA-protein pull-down assays were performed to detect and identify the AFAP1-AS1-interacting protein KAT2B. Protein-RNA immunoprecipitation (RIP) assays were conducted to examine the interaction of AFAP1-AS1 and KAT2B. Chromatin immunoprecipitation (ChIP) and luciferase analyses were utilized to identify the binding site of transcription intermediary factor 1 alpha (TIF1α) and H3K14ac on the RBM3 promoter.

**Results:**

AFAP1-AS1 is upregulated in NPC and is a poor prognostic indicator for survival in NPC patients. AFAP1-AS1 was required for NPC proliferation *in vitro* and tumorigenicity *in vivo*. Mechanistic investigations suggested that AFAP1-AS1 binds to KAT2B and promotes acetyltransferase activation at two residues (E570/D610). KAT2B further promotes H3K14 acetylation and protein binding to the bromo domain of TIF1α. Consequently, TIF1α acts as a nuclear transcriptional coactivator of RBM3 transcription, leading to YAP mRNA stabilization and enhanced NPC tumorigenicity.

**Conclusions:**

Our findings suggest that AFAP1-AS1 functions as an oncogenic biomarker and promotes NPC tumorigenicity through enhanced KAT2B acetyltransferase activation and YAP mRNA stabilization.

## Introduction

Nasopharyngeal carcinoma (NPC), a common head and neck malignancy, has a high incidence rate in Southern China (1–3). Although radiotherapy is one of the most effective treatments for NPC patients (4, 5), recurrence and local advancement often lead to treatment failure. Therefore, understanding the potential molecular mechanisms underlying NPC pathogenesis is urgent. The identification of novel therapeutic targets can help in the development of new therapeutic programs that will improve the overall survival rates of patients with NPC. Increasing evidence suggests that long noncoding RNAs (lncRNAs) play important roles in NPC tumorigenicity (6–8). However, the mechanisms through which NPC tumorigenicity is regulated remain poorly understood.

Actin filament associated protein 1 antisense RNA1 (AFAP1-AS1) is an oncogenic lncRNA associated with the pathogenesis of a variety of cancers. AFAP1-AS1 was first mapped to the antisense strand of AFAP1 DNA (9). Studies have shown that AFAP1-AS1 is upregulated in esophageal cancer (9), lung cancer (10), hepatocellular carcinoma (11), pancreatic cancer (12), colorectal cancer (13), and nasopharyngeal carcinoma (14). Recently, it was shown that higher AFAP1-AS1 expression levels are associated with a poor prognosis in NCP patients (15, 16). Recent evidence suggests that AFAP1-AS1 binds to and promotes EZH2 methyltransferase activity in colorectal cancer (13). AFAP1-AS1 has also been linked to regulation of the Rho/Rac pathway *via* competition with endogenous miR-423-5p (14). Interestingly, AFAP1-AS1 has been shown to influence both KRT1 and AFAP1 expression *via* both trans- and cis-regulatory mechanisms (17). Furthermore, AFAP1-AS1 was also shown to be a direct downstream gene target for c-Myc (18). However, the function of AFAP1-AS1 in NPC tumorigenicity remains largely unknown.

In this study, we used RNA-Seq and liquid chromatography–tandem mass spectrometry (LC-MS/MS) assays to evaluate NPC cell lines. Through our study, we identified a new molecular mechanism underlying the role of AFAP1-AS1 in NPC tumorigenicity. We found that AFAP1-AS1 enhances KAT2B acetyltransferase activation, which upregulates H3K14ac activity against TIF1α, resulting in enhanced RBM3 transcription and subsequent stabilization of YAP mRNA, inducing AFAP1-AS1-driven NPC tumorigenicity.

## Materials and Methods

### Clinical Specimens

Ten pairs of freshly frozen NPC tumor and adjacent nontumor specimens were obtained from Zhongshan City People’s Hospital. We also collected an additional 100 pairs of formalin-fixed paraffin-embedded NPC tumor and adjacent nontumor specimens from Zhengjiang Provincial People’s Hospital. None of the samples were collected from patients undergoing chemo- or radiotherapy at the time of biopsy. Written ethics consent (No:2020QT254) was obtained from the Institutional Ethical Review Board of the Zhengjiang Provincial People’s Hospital before the samples were analyzed.

### Cell Culture

HNE-1, C666-1, SUNE-1, CNE-1, CNE-2, and NP69 cell lines were all procured from the Cell Bank of the Chinese Scientific Academy (Shanghai, China). NPC cell lines were maintained in Dulbecco’s Modified Eagle’s Medium (DMEM) supplemented with 10% fetal bovine serum. NP69 was cultured in DMEM/F12 supplemented with epidermal growth factor (20 ng/mL), cholera toxin (100 ng/mL), insulin (10 μg/mL), penicillin–streptomycin (100 μg/mL), hydrocortisone (0.5 μg/mL), and horse serum (5%). All cells were cultured in an incubator with CO_2_ (5%) at 37°C. STR DNA fingerprinting was performed by the Shanghai Biowing Applied Biotechnology Co., Ltd. (Shanghai, China) to confirm identity before the start of the study.

### Quantitative Reverse Transcriptase PCR Analysis

Quantitative reverse transcriptase PCR (qRT-PCR) was performed as previously described (6). Briefly, total RNA was isolated from NPC cell lines and the collected specimens using TRIzol reagent (Invitrogen), followed by reverse transcription using reverse transcriptase (Promega). Finally, the QuantiTect SYBR Green PCR Kit (Thermo Fisher) was used to perform qRT-PCR. mRNA quantification was normalized to β-actin. All the primers used in this study are listed in **Supplementary Table 1**.

### Immunoprecipitation and Western Blotting Assays

Immunoprecipitation (IP) and Western blotting (WB) assays were conducted as previously described (19). The specific antibodies used were as follows: KAT2B (#3378, 1:1000, Cell Signaling Technology), H3K9ac (#9649, 1:1000, Cell Signaling Technology), H3K14ac (#26828, 1:1000, Cell Signaling Technology), H3K9ac (#9649, 1:1000, Cell Signaling Technology), Histone H3 (#4499, 1:1000, Cell Signaling Technology), TIF1α (ab38264, 1:1000, Abcam), TIF1β (#4124, 1:1000, Cell Signaling Technology), TIF1γ (#13387, 1:1000, Cell Signaling Technology), NCOA1 (#20301, 1:1000, Cell Signaling Technology), NCOA2 (#96687, 1:1000, Cell Signaling Technology), NCOA3 (#2126, 1:1000, Cell Signaling Technology), RBM3 (ab211356, 1:1000, Abcam), YAP (#14074, 1:1000, Cell Signaling Technology), Flag M2 antibody (#14793, 1:1000, Cell Signaling Technology), HA antibody (#2367, 1:1000, Cell Signaling Technology), and β-actin (#4970, 1:1000, Cell Signaling Technology).

### Colony Formation and Cell Proliferation Assays

To perform the colony formation assay, cells were seeded into six-well plates with DMEM supplemented with 10% fetal bovine serum and cultured (as described in the “Cell Culture” section) for approximately 10 days. The cell colonies were then fixed, stained, and counted. For the cell proliferation assays, cells were seeded into DMEM containing 10% fetal bovine serum, passaged, and finally detected using a WST-1 assay kit.

### RNA Pull-Down and RNA Immunoprecipitation Assays

RNA pull-down and RNA immunoprecipitation (RIP) assays were performed as previously shown (20). The specific antibodies used were as follows: KAT2B (#3378, 1:1000, Cell Signaling Technology) and Flag M2 antibody (#14793, 1:1000, Cell Signaling Technology).

### Chromatin Immunoprecipitation-qPCR

We first performed chromatin immunoprecipitation (ChIP) using the Chromatin Immunoprecipitation Kit (Millipore-17-408) in accordance with the manufacturer’s instructions. Then, we purified the immunoprecipitated DNA using the phenol extraction method and quantified the relevant DNA using qPCR. All the primers used in this study are listed in **Supplementary Table 1**.

### Promoter Reporter and Dual-Luciferase Assays

The RBM3 promoter was amplified from NP69 cells using PCR and then subcloned into an empty pGL3 vector. Renilla luciferase reporter plasmid was also transfected as a normalization control. Dual-luciferase assays were performed using a dual-luciferase assay kit (#E1910, Promega) according to the manufacturer’s instructions. The details of the primers used to clone the RBM3 promoter are described in **Supplementary Table 1**.

### In Situ Hybridization

In situ hybridization analysis of AFAP1-AS1 interactions was performed on paraffin-embedded sections using the RNAscope 2.5 HD Detection Reagent-BROWN kit (ACDBio). All analyses were carried out according to the manufacturer’s instructions using an AFAP1-AS1–specific probe purchased from ACDBio.

### Plasmids

AFAP1-AS1, KAT2B, TIF1α, and RBM3 transcripts from NP69 cells were amplified using RT-PCR, sequenced, and then subcloned into the pcDNA3.0 or pLVX-Puro vector (Clontech). AFAP1-AS1 or TIF1α-truncated constructs were generated using PCR with AFAP1-AS1 or TIF1α-pcDNA3.0 as templates and inserted into pcDNA3.0. KAT2B^E570A/D610A^ and TIF1α^F979A/N980A^ point mutations were generated using a PCR-based site-directed mutagenesis kit from Invitrogen following the manufacturer’s instructions.

### shRNA Knockdown and Transfection

shRNA-knockdown and transfection assays were performed as previously described (21). shRNA sequences were purchased from Shanghai Biogene Co., Ltd. (Shanghai, China). These sequences and the packaging plasmids were transfected into HEK293 cells and used to generate viral particles. The supernatants were collected at 48 and 72 hours posttransfection and filtered through a 0.22 µm membrane (Millipore). The viruses were then concentrated. HNE-1 and CNE-2 cells were infected with these shRNA or shGFP control viruses in the presence of 8 µg/mL polybrene. Infected cells were enriched by selection with 5 μg/mL puromycin 48 hours after infection. Multiple monoclonal cultures were screened for shRNA integration and activity using WB and RT-PCR.

### RNA-Seq and Differentially Expressed Gene Analysis

RNA-Seq and differentially expressed gene analysis were performed as previously described (6). RNA-Seq data generated in this study are available from the NCBI BioProject database (http://www.ncbi.nlm.nih.gov/bioproject) under Bioproject ID: PRJNA594347.

### Mass Spectrometry of AFAP1-AS1-Associated Proteins

AFAP1-AS1 and its antisense plasmid were cut, transcribed, and biotin-labeled *in vitro* with Bio16-UTP (Life Technologies) using a MAXIscript T7 Transcription Kit (Life Technologies). Protein–RNA interactions in HNE-1 cell lysates were analyzed using a Pierce Magnetic RNA-Protein Pull-Down Kit (Life Technologies). The retrieved proteins were then subjected to WB analysis or resolved by gradient gel electrophoresis and subjected to LC-MS/MS sequencing and data analysis.

### Immunohistochemical Staining

Immunohistochemistry (IHC) was performed using an anti-KAT2B antibody (#3378, 1:1000, Cell Signaling Technology). Each sample was assigned a score according to the intensity of the KAT2B staining (0 = no staining, 1 = weak staining, 2 = moderate staining, and 3 = strong staining) and the proportion of stained cells (0 = 0%, 1 = 1%–25%, 2 = 25%–50%, 3 = 50%–75%, 4 = 75%–100%). Negative control slides without primary antibodies were included as a reference. The core staining was scored as negative (0) when <10% tumor cells showed KAT2B expression. The stained tissues were scored by three individuals blinded to the clinical parameters.

### Tumorigenesis Studies

To evaluate NPC tumorigenicity, four-week-old female BALB/c nude mice, maintained in a mouse-specific pathogen-free (SPF) facility, were injected subcutaneously with HNE-1 cells (2 × 10^6^). All experimental procedures were performed in accordance with the Animal Care and Use Committee of Chinese Academy of Medical Science guidelines.

### Statistics

All statistical analyses were performed using GraphPad Prism version 5.0 software. Survival rates were determined using the Kaplan-Meier method, and significance was determined using multiple comparison tests. A *P* value < 0.05 was considered statistically significant.

## Results

### lncRNA AFAP1-AS1 Expression Is a Biomarker of Poor Prognosis in NPC Patients

**Figure 1:**
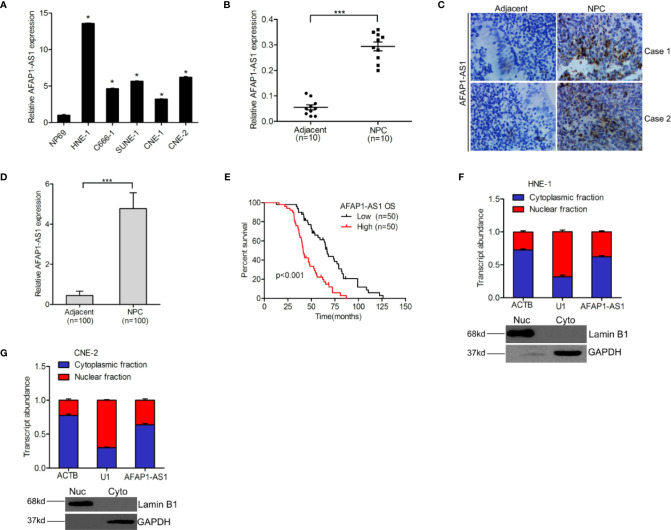
lncRNA AFAP1-AS1 is a marker for poor prognostic outcomes in NPC patients. **(A)** AFAP1-AS1 expression in NPC cells and nasopharyngeal epithelial cells. **(B)** Compared with paired nontumor specimens, AFAP1-AS1 expression is upregulated in freshly frozen NPC tumor tissues. **(C)** Representative images of AFAP1-AS1 expression in paraffin-embedded NPC tumors and paired adjacent nontumor specimens detected using RNAscope. Scale bars: 50 µm. **(D)** Quantification of AFAP1-AS1 expression in **(C)**. **(E)** Kaplan-Meier analysis of overall survival (OS) in NPC patients with high vs. low AFAP1-AS1 expression. **(F, G)** AFAP1-AS1 is expressed in both the nucleus and cytoplasm of NPC cells as detected using qRT-PCR. Error bars represent the standard deviation for each value. *P < 0.05. ***P < 0.001. The data represent three independent experiments. lncRNA, long noncoding RNA.

To determine the roles of AFAP1-AS1 in NPC progression, we first evaluated changes in AFAP1-AS1 expression in HNE-1, C666-1, SUNE-1, CNE-1, CNE-2 NPC, and NP69 nasopharyngeal epithelial cells. As shown in **Figure 1A**, AFAP1-AS1 expression was higher in all five NPC cell lines than in the NP69 control, and its expression was the highest in HNE-1 and CNE-2 cells. To elaborate on these results, we examined AFAP1-AS1 expression in 10 pairs of freshly frozen NPC tumor and adjacent nontumor specimens using qRT-PCR. Interestingly, AFAP1-AS1 expression was significantly higher in NPC tumors than in their adjacent nontumor pair (**Figure 1B**). RNA *in situ* hybridization was then used to analyze AFAP1-AS1 expression in 100 pairs of paraffin-embedded NPC tumor and adjacent nontumor specimens, which revealed that AFAP1-AS1 expression was higher in NPC tumors (**Figures 1C, D**), confirming the aforementioned qRT-PCR result.

To reveal the clinical significance of AFAP1-AS1 expression in NPC, we analyzed the correlation between AFAP1-AS1 expression and NPC patient survival rates. Kaplan-Meier survival assays (**Figure 1E**) demonstrated that NPC patients with higher AFAP1-AS1 expression levels had lower overall survival rates than patients with lower AFAP1-AS1 expression levels. Additionally, AFAP1-AS1 was shown to be expressed in both the nucleus and cytoplasm of NPC (**Figures 1C, F, G**), suggesting that AFAP1-AS1 may have multiple functions in both the nucleus and cytoplasm of tumor cells. In conclusion, these data suggest that AFAP1-AS1 expression is a good biomarker for the prognosis of NPC patients.

### AFAP1-AS1 Expression Is Important for NPC Growth and Tumorigenicity

**Figure 2:**
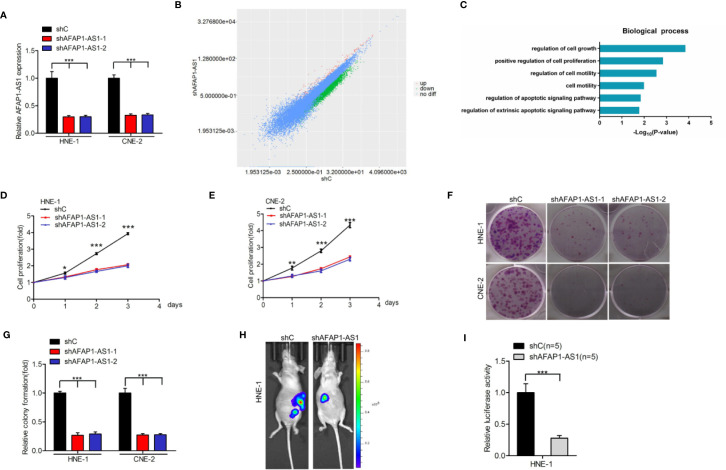
AFAP1-AS1 is important for NPC growth and tumorigenicity. **(A)** qRT-PCR analysis of AFAP1-AS1 knockdown by two shRNAs (shAFAP1-AS1-1 and shAFAP1-AS1-2) vs. a control shRNA (shC). **(B)** Scatter plot of gene expression in HNE-1 cells transfected with AFAP1-AS1 shRNA (y-axis) compared with the control (x-axis). Green dots, genes that are significantly downregulated following AFAP1-AS1 knockdown. Red dots, genes that are significantly upregulated following AFAP1-AS1 knockdown. Black dots, genes with no change in expression following AFAP1-AS1 knockdown. **(C)** GO analyses of AFAP1-AS1 knockdown-associated biological process signaling pathways. **(D, E)** AFAP1-AS1 depletion-inhibited cellular proliferation in HNE-1 and CNE-2 cells. **(F)** Effects of AFAP1-AS1 depletion on cell colony formation. **(G)** Quantification of colony formation in **(F)**. **(H)** Representative bioluminescence images following AFAP1-AS1 depletion suppressing HNE-1 subcutaneous tumor growth. **(I)** Quantification of the bioluminescence activity in **(H)**. Error bars represent standard deviation. *P < 0.05. **P < 0.01. ***P < 0.001. The data represent three independent experiments. GO, gene ontology.

To reveal whether AFAP1-AS1 is important for NPC tumorigenicity, we first depleted endogenous AFAP1-AS1 using two different shRNAs in both HNE-1 and CNE-2 cell lines (**Figure 2A**). We then performed RNA-Seq to compare mRNA expression profiles. Several differentially expressed genes (DEGs) were identified (*P* < 0.05 and a fold change > 1.5) in AFAP1-AS1 knockdown-inhibited HNE-1 cells (**Figure 2B**). These DEGs were then subjected to gene ontology (GO) analysis (**Figure 2C**), which revealed that the majority of enrichment occurred in the cellular proliferation pathways. These data suggest that AFAP1-AS1 may play a vital role in NPC proliferation.

To confirm the results of the RNA-Seq data, we evaluated the effects of AFAP1-AS1 depletion using cellular proliferation (**Figures 2D, E**) and colony formation (**Figures 2F, G**) assays in both HNE-1 and CNE-2 cells. AFAP1-AS1 knockdown markedly reduced HNE-1 tumorigenicity (**Figures 2H, I**). In contrast, AFAP1-AS1 overexpression promoted cellular proliferation and colony formation in NP69 cells (**Supplementary Figure 1**). Collectively, these data suggest that AFAP1-AS1 is important for NPC growth and tumorigenicity, and may be important in the transition of normal cells to tumor precursors.

### AFAP1-AS1 Enhances NPC Cellular Proliferation *via* YAP

**Figure 3:**
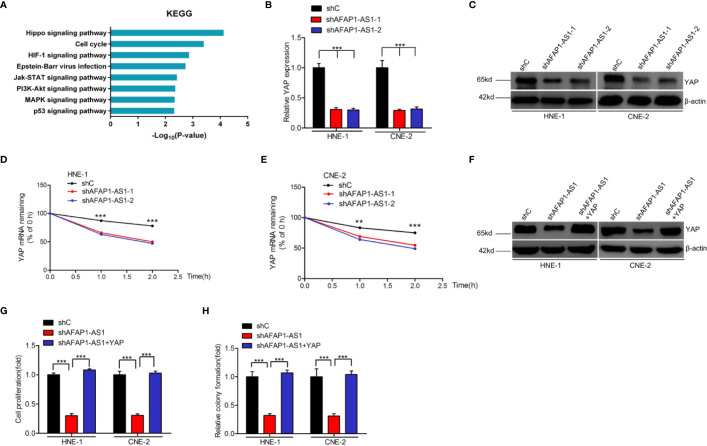
AFAP1-AS1 enhances NPC cell proliferation *via* YAP stabilization. **(A)** KEGG analyses of AFAP1-AS1 knockdown-associated signaling pathways. **(B, C)** Effects of AFAP1-AS1 depletion on YAP mRNA **(B)** and protein **(C)** expression. **(D, E)** Effects of reduced YAP mRNA stability in AFAP1-AS1–depleted HNE-1 cells (compared to the control). Cells were treated with 1 μg/mL actinomycin D, and RNA was extracted at 0, 1, and 2 hours. **(F–H)** YAP rescued AFAP1-AS1 depletion-inhibited YAP expression **(F)**, cellular proliferation **(G)**, and colony formation **(H)**. Error bars represent standard deviation. **P < 0.01. ***P < 0.001. The data represent three independent experiments.

To understand the potential mechanisms by which AFAP1-AS1 enhances cellular proliferation, we first conducted Kyoto Encyclopedia of Genes and Genomes (KEGG) analysis. This analysis revealed that the Hippo signaling pathway was one of the top differentially changed AFAP1-AS1–induced pathways (**Figure 3A**). Furthermore, both mRNA and protein expression of YAP, a vital regulator of the Hippo pathway, were inhibited by AFAP1-AS1 depletion (**Figures 3B, C**), suggesting that YAP may be a critical component of the AFAP1-AS1-mediated network. Moreover, we found AFAP1-AS1 knockdown inhibited YAP expression *in vivo* (**Supplementary Figure 2**).

Interestingly, we found that YAP mRNA degraded faster upon AFAP1-AS1 knockdown in HNE-1 cells, which were treated with the RNA synthesis inhibitor actinomycin D, and analyzed at 0, 1, and 2 hours post-exposure (**Figures 3D, E**). These results indicate that AFAP1-AS1 may play a vital role in YAP mRNA stability.

To assess the role of YAP in AFAP1-AS1-regulated NPC proliferation, we overexpressed YAP in AFAP1-AS1–depleted HNE-1 and CNE-2 cells (**Figure 3F**). Exogenous YAP overexpression rescued AFAP1-AS1 depletion-inhibited cell proliferation (**Figure 3G**) and colony formation (**Figure 3H**). Taken together, these results reveal that YAP is a downstream effector of AFAP1-AS1–mediated NPC proliferation.

### AFAP1-AS1 Physically Binds to KAT2B

**Figure 4:**
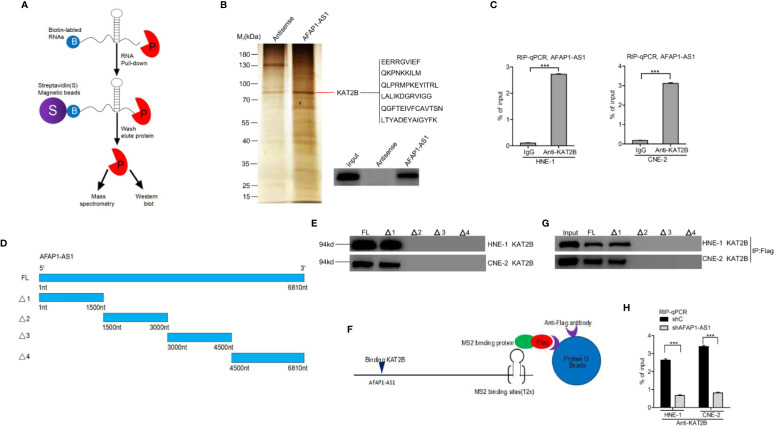
AFAP1-AS1 physically binds to KAT2B. **(A)** Schematic description of the RNA pull-down assay for the identification of AFAP1-AS1–associated proteins. **(B)** Representative image of silver staining, revealing AFAP1-AS1–associated proteins in HNE-1 cells. **(C)** RIP assay showing that AFAP1-AS binds to KAT2B. **(D)** Schematics of full-length AFAP1-AS1 and various AFAP1-AS1 deletion mutants. **(E)** Effects of AFAP1-AS1 mutations on **(D)**. KAT2B binding evaluated by an RNA pull-down assay. **(F, G)** Western blot detection of KAT2B binding of AFAP1-AS1–truncated constructs using a Flag-MS2bp-MS2bs–based pull-down assay. **(H)** Effects of AFAP1-AS1 depletion on AFAP1-AS1 binding of KAT2B. Error bars represent standard deviation. ***P < 0.001. The data represent three independent experiments. RIP, RNA immunoprecipitation.

Increasing evidence suggests that lncRNAs can exert their function through binding histone acetyltransferase proteins, including the KAT family proteins (6, 22). We hypothesized that AFAP1-AS1–mediated proliferation may rely on its association with certain KAT family proteins. As shown in **Figures 4A**, **B**, we used biotin-labeled RNA pull-down and mass spectrometry to establish that KAT2B was significantly associated with AFAP1-AS1. Thus, we selected KAT2B as the effector protein for all our downstream analyses. First, we validated the AFAP1-AS1/KAT2B association using an RNA pull-down and RNA Immunoprecipitation assay (**Figures 4B, C**), which also confirmed that AFAP1-AS1 did not bind to other KAT family proteins (KAT2A, KAT3A, KAT3B, KAT6A, KAT6B, KAT7, KAT8) (**Supplementary Figure 3A**). KAT2B is also a proven oncogene that activates gene transcription in medulloblastoma and glioblastoma (23). Second, we found that KAT2B was higher in NPC tumor tissues than in their adjacent nontumor tissues (**Supplementary Figure 3B, C**). Taken together, these data indicate that AFAP1-AS1 directly binds to KAT2B, which then modulates histone modification.

To further validate these findings, we performed deletion mapping followed by a pull-down to reveal KAT2B associations with specific regions of AFAP1-AS1 (**Figures 4D, E**). As shown in **Figure 4E**, a region covering 0–1500 nucleotides at the 5′ end of AFAP1-AS1 is critical for KAT2B binding. A Flag-MS2bp-MS2bs system used in conjunction with immunoprecipitation assays demonstrated similar results (**Figures 4F, G**), suggesting that this was a valid observation and not the result of the assay design. Finally, AFAP1-AS1 depletion inhibited the KAT2B association in RIP qRT-PCR assays (**Figure 4H**).

### AFAP1-AS1 Promotes YAP mRNA Stability *via* KAT2B

**Figure 5:**
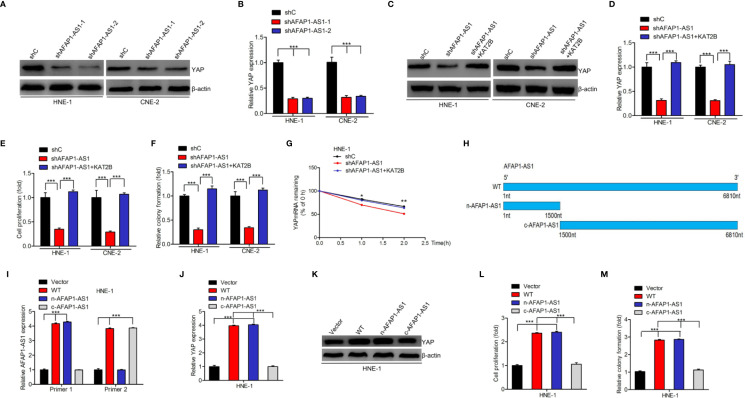
AFAP1-AS1 promotes YAP mRNA stability *via* KAT2B. **(A, B)** AFAP1-AS1 depletion inhibited YAP protein **(A)** and mRNA **(B)** expression. **(C–G)** Effects of KAT2B overexpression on AFAP1-AS1 depletion: inhibited YAP protein expression **(C)**, YAP mRNA expression **(D)**, cellular proliferation **(E)**, colony formation **(F)**, and YAP mRNA stability **(G)**. **(H)** Schematics of full-length AFAP1-AS1 and AFAP1-AS1 deletion mutants at the nitrogen (N) or carbon **(C)** protein sites. **(I)** Effects of AFAP1-AS1 plasmids in **(H)** overexpressing HNE-1 cells. **(J–M)** Effects of AFAP1-AS1 plasmid in **(H)** overexpressing cells on YAP mRNA expression **(J)**, YAP protein expression **(K)**, cellular proliferation **(L)**, and colony formation (M). Error bars represent the standard deviation. *P < 0.05. **P < 0.01. ***P < 0.001. The data represent three independent experiments.

As YAP is an important downstream effector of AFAP1-AS1 and since AFAP1-AS1 is bound to KAT2B, we hypothesized that AFAP1-AS1 promotes YAP mRNA stability *via* KAT2B regulation in NPC cells. We first downregulated AFAP1-AS1 expression and found that both YAP mRNA and protein expression were also inhibited (**Figures 5A, B**). Moreover, KAT2B overexpression restored AFAP1-AS1 depletion-inhibited YAP protein (**Figure 5C**) and mRNA expression (**Figure 5D**) as well as improved its mRNA stability (**Figure 5G**) in NPC cells. Both cellular proliferation and colony formation were also rescued following KAT2B overexpression in AFAP1-AS1–depleted HNE-1 and CNE-2 cells (**Figures 5E, F**).

To evaluate the mechanisms underlying the AFAP1-AS1/KAT2B/YAP associations, we constructed full length AFAP1-AS1 WT, n-AFAP1-AS1 mutant, and c-AFAP1-AS1 mutant plasmids (**Figure 5H**) and designed specific qRT-PCR primers to detect their transcripts (**Figure 5I**). As shown in **Figure 5J–M**, n-AFAP1-AS1, which contains the AFAP1-AS1/KAT2B association domains, promoted YAP mRNA and protein expression, cell proliferation, and colony formation. The same was not true for the c-AFAP1-AS1 mutant, which lacked this functional domain. Taken together, our results suggest that AFAP1-AS1 promotes YAP mRNA stability by binding to KAT2B.

### KAT2B Acetyltransferase Activity Promotes AFAP1-AS1–Induced Stabilization of YAP

**Figure 6:**
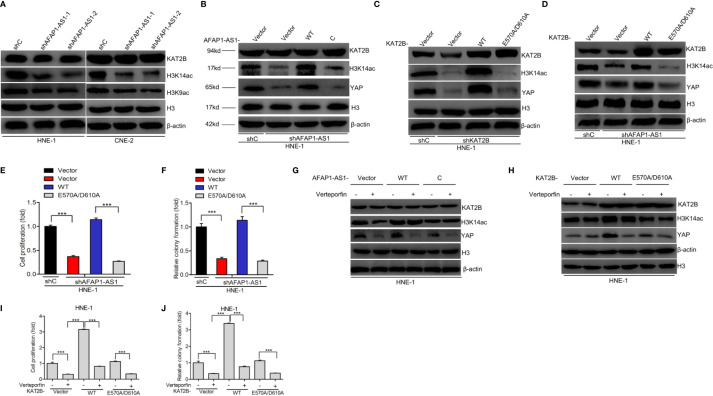
KAT2B acetyltransferase activity promotes AFAP1-AS1 stabilization of YAP. **(A)** Effects of AFAP1-AS1 depletion on the acetylation of H3K9 and H3K14 and KAT2B expression. **(B)** Overexpression of AFAP1-AS1 wild-type, but not c-AFAP1-AS1 deletion mutants, rescues AFAP1-AS1 depletion-suppressed YAP expression. **(C)** Effects of overexpression of KAT2B wild-type and acetyltransferase activity-deficient mutant, E570A/D610A, on KAT2B depletion-suppressed YAP expression. **(D–F)** Overexpression of KAT2B wild-type, but not the acetyltransferase activity-deficient mutant E570A/D610A, restored AFAP1-AS1 depletion-suppressed YAP expression **(D)**, cellular proliferation **(E)**, and colony formation **(F)**. **(G)** Inhibition of YAP reduced AFAP1-AS1–enhanced YAP protein expression. **(H–J)** Effects of YAP inhibition on KAT2B-enhanced YAP protein expression **(H)**, cellular proliferation **(I)**, and colony formation **(J)**. Error bars represent standard deviation. ***P < 0.001. The data represent three independent experiments.

As AFAP1-AS1 stabilized YAP mRNA by binding to KAT2B, we further explored the potential molecular mechanisms underlying this observation. To do this, we evaluated histone H3 acetylation levels in NPC cell lines. We found that the acetylated levels of H3K9 and H3K14, the proven targets of KAT2B (24), were upregulated in the presence of AFAP1-AS1. As shown in **Figure 6A**, AFAP1-AS1 knockdown decreased H3K9 and H3K14 acetylation, but did not impact KAT2B protein expression. When histone H3 acetylation marks were compared, H3K14 acetylation levels were particularly inhibited by AFAP1-AS1 depletion in both HNE-1 and CNE-2 cells (**Figure 6A**). These results indicate that AFAP1-AS1 may promote KAT2B acetyltransferase activity.

As the roles of H3K14 acetylation modulated by AFAP1-AS1 in NPC remain poorly understood, we sought to determine how H3K14ac modulation affects the function of AFAP1-AS1–regulated NPC cell proliferation. To assess whether KAT2B acetyltransferase activity promoted the function of AFAP1-AS1–stabilized YAP, we created a KAT2B acetyltransferase activity-deficient mutant, KAT2A^E570A/D601A^ (25), and checked whether AFAP1-AS1 could bind to these KAT2B peptides (**Figure 4B**). Next, we hypothesized that the E570/D610 residues of KAT2B were critical for AFAP1-AS1–driven YAP stabilization. As shown in **Figure 6B**, n-AFAP1-AS1, but not c-AFAP1-AS1, mutants could rescue AFAP1-AS1 depletion-inhibited YAP expression. Exogenous KAT2B^WT^, but not the KAT2B^E570A/D601A^ mutant, restored KAT2B depletion-regulated changes in YAP expression (**Figure 6C**). Furthermore, YAP expression (**Figure 6D**), cell proliferation (**Figure 6E**), and colony formation (**Figure 6F**) abilities in AFAP1-AS1–depleted HNE-1 cells were suppressed by the overexpression of KAT2B^WT^, but not the KAT2B^E570A/D601A^ mutant. These data support our hypothesis that residues E570/D610 are required for AFAP1-AS1–modulated YAP stabilization.

Next, we evaluated whether the YAP inhibitor verteporfin mediates AFAP1-AS1–induced or KAT2B-induced NPC cellular proliferation. As shown in **Figure 6G**, verteporfin significantly decreased YAP expression in AFAP1-AS1-WT–overexpressed HNE-1 cells compared with that in c-AFAP1-AS1 or empty vector-overexpressing HNE-1 cells. We then observed that verteporfin markedly suppressed YAP expression (**Figure 6H**), cellular proliferation (**Figure 6I**), and colony formation (**Figure 6J**) in HNE-1 cells transfected with KAT2B^WT^ compared with cells transfected with KAT2B^E570A/D601A^ or empty vector controls. This led us to conclude that KAT2B acetyltransferase activity promotes AFAP1-AS1–mediated stabilization of YAP and cellular proliferation.

### Binding of TIF1α to H3K14ac Is Required for AFAP1-AS1–Driven YAP Stabilization

**Figure 7:**
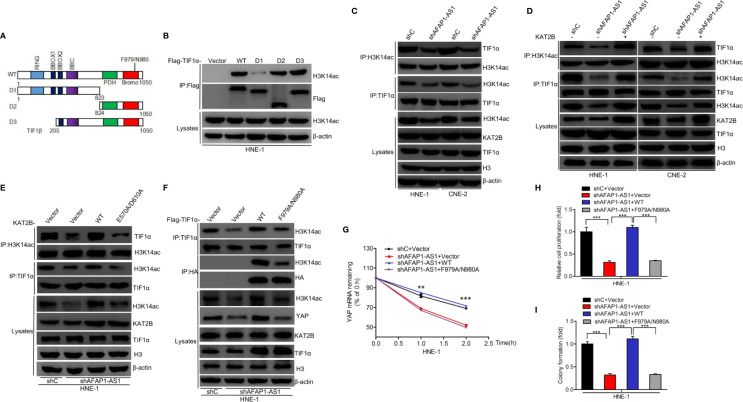
Binding of H3K14ac to TIF1α is required for AFAP1-AS1–driven YAP stabilization. **(A)** Schematics of TIF1α^WT^ and various TIF1α deletion mutants. **(B)** H3K14ac interacts with TIF1α through amino acid residues 824–1050. **(C)** AFAP1-AS1 depletion inhibited TIF1α interaction with H3K14ac. **(D)** Effects of KAT2B overexpression on AFAP1-AS1 depletion-inhibited TIF1α interaction with H3K14ac. **(E)** Effects of KAT2B wild-type and E570A/D610A mutant overexpression on AFAP1-AS1 knockdown suppressed the binding of H3K14ac to TIF1α. **(F–I)** Overexpression of TIF1α wild-type and F979A/N980A mutant restored AFAP1-AS1 knockdown- suppressed binding of H3K14ac with TIF1α **(F)**, YAP mRNA stability **(G)**, cellular proliferation **(H)**, and colony formation **(I)**. Error bars represent standard deviation. **P < 0.01. ***P < 0.001. The data represent three independent experiments.

Recent evidence has shown that H3 acetylation of the transcription intermediary factor (TIF) family of proteins is important for cell proliferation in a number of different cancers (26–29). We hypothesized that KAT2B enhanced H3K14 acetylation of TIF proteins, which then acted as transcriptional activators enhancing downstream gene expression, resulting in improved YAP stability and promoting NPC proliferation. To validate this hypothesis, we used immunoprecipitation to show that TIF1α, TIF1β, and TIF1γ, but not NCOA1, NCOA2, or NCOA3, bind to H3K14ac (**Supplementary Figure 4A**). Further investigations revealed that TIF1α, but not TIF1β or TIF1γ, rescued AFAP1-AS1 depletion-inhibited YAP mRNA stability (**Supplementary Figure 4B**). Thus, we focused on TIF1α for further investigation. To determine which region of TIF1α is critical for the TIF1α association with H3K14ac, we created deletion mutants removing various functional regions of the protein (**Figure 7A**) and then transfected them into HNE-1 cells. As shown in **Figure 7B**, D2 and D3 mutants, but not the D1 mutant, without PHD and bromodomain domains could not associate with H3K14ac. This demonstrated that amino acids 824–1050 in TIF1α are critical for the interaction between TIF1α and H3K14ac. Next, we revealed that AFAP1-AS1 depletion suppressed the TIF1α association with H3K14ac, while exogenous KAT2B expression restored it (**Figures 7C, D**). We also found that KAT2B^WT^ overexpression restored AFAP1-AS1–mediated interference between TIF1α and H3K14ac binding, but that KAT2B^E570A/D601A^ overexpression could not (**Figure 7E**). A recent study reported that residues F979/N980 in TIF1α are required for its association with H3K9ac (23), and we hypothesized that TIF1α binds to H3K14ac *via* F979/N980. To test this hypothesis, we generated one TIF1α^F979A/N980A^ mutant in which the binding between H3K14ac and TIF1α was disrupted. As shown in **Figure 7F**, overexpression of TIF1α^WT^ rescued the AFAP1-AS1–mediated loss of TIF1α associated with H3K14ac, while overexpression of TIF1α^F979A/N980A^ did not. Moreover, TIF1α^WT^ overexpression, but not TIF1α^F979A/N980A^ overexpression, restored AFAP1-AS1 depletion-suppressed YAP mRNA stability (**Figure 7G**), cellular proliferation (**Figure 7H**), and colony formation (**Figure 7I**) in HNE-1 cells. Taken together, our results suggest that the interaction between TIF1α and H3K14ac is crucial for AFAP1-AS1–driven YAP stabilization.

### TIF1α/H3K14ac Complex-Activated RBM3 Transcription Is Required for the AFAP1-AS1 Modulation of YAP Stability

**Figure 8:**
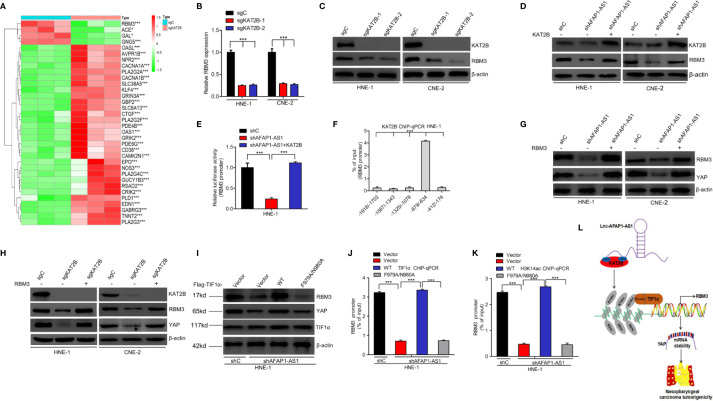
TIF1α/H3K14ac complex activation of RBM3 transcription is required for AFAP1-AS1–modulation of YAP stability. **(A)** Heatmaps of RNA-Seq data for RBM3 and its target genes in HNE-1 cells with KAT2B sgRNA or a control sgRNA. **(B, C)**, KAT2B knockout inhibits RBM3 mRNA **(B)** and protein **(C)** expression. **(D, E)** Effects of KAT2B overexpression on AFAP1-AS1 knockdown-suppressed RBM3 protein expression **(D)** and RBM3 promoter activity **(E)**. **(F)** ChIP-qPCR assay of KAT2B binding at different loci within the RBM3 promoter. IgG is used as a control. **(G)** RBM3 overexpression rescues AFAP1-AS1 knockdown-mediated suppression of YAP. **(H)** Effects of RBM3 overexpression on KAT2B knockout-mediated YAP repression. **(I)** TIF1α wild-type, but not the F979A/N980A mutant, restores AFAP1-AS1 knockdown-suppressed YAP expression in HNE-1 cells. **(J, K)** Effect of TIF1α wild-type and F979A/N980A mutant overexpression on AFAP1-AS1 knockdown-suppressed TIF1α binding **(J)**, H3K14ac **(K)**, and the RBM3 promoter. **(L)** A working model for AFAP1-AS1–mediated NPC tumorigenicity. Error bars represent the standard deviation. *P < 0.05. ***P < 0.001.The data represent three independent experiment.

Heatmaps of the RNA-Seq analysis revealed that RBM3 and its downstream genes (28) were significantly affected by KAT2B deletion (**Figure 8A**). RBM3 has been shown to stabilize YAP mRNA expression during cold stress (30). We hypothesized that activation of RBM3 transcription by the TIF1α/H3K14ac complex might be required for AFAP1-AS1 modulation of YAP stability. As shown in **Figures 8B**, **C**, KAT2B deletion markedly decreased RBM3 mRNA and protein expression. Overexpression of KAT2B restored AFAP1-AS1 knockdown-inhibited RBM3 expression (**Figure 8D**). We also determined that KAT2B bound to the RBM3 promoter at –879 to –634 bp (**Figure 8F**), and KAT2B overexpression restored the promoter activity of RBM3 suppressed by AFAP1-AS1 knockdown (**Figure 8E**). Knockdown of RBM3 suppressed YAP protein expression, mRNA expression, and mRNA stability (**Supplementary Figure 5**). Moreover, RBM3 overexpression restored AFAP1-AS1 knockdown or KAT2B knockout-suppressed YAP expression (**Figures 8G, H**). We further found that overexpression of TIF1α^WT^ restored the loss of YAP expression caused by AFAP1-AS1 depletion, but overexpression of TIF1α^F979A/N980A^ did not alter the YAP expression profile (**Figure 8I**). Consistent with these results, exogenous TIF1α^WT^ expression promoted TIF1α/H3K14ac binding to the RBM3 promoter, while exogenous expression of TIF1α^F979A/N980A^ did not (**Figures 8J, K**). These data support our conclusion that activation of RBM3 transcription by the TIF1α/H3K14ac complex is critical for AFAP1-AS1–mediated YAP stability.

## Discussion

Our results show that AFAP1-AS1 binds to KAT2B and induces KAT2B acetyltransferase activity in NPC cells. KAT2B-enhanced H3K14ac, in turn, binds to TIF1α, leading to the upregulation of RBM3 transcription, YAP mRNA stability, and increased NPC tumorigenicity (**Figure 8L**).

Our results also suggest that AFAP1-AS1 acts as an oncogene in NPC, which is in accordance with the results of previous studies (14–16). Additionally, AFAP1-AS1 expression was upregulated in NPC tissues, and the high level of expression was negatively associated with NPC survival prognosis. The results of the RNA-Seq data analysis show that AFAP1-AS1 is positively correlated with cellular proliferation-associated pathways. Moreover, AFAP1-AS1 is required for NPC cell proliferation and colony formation *in vitro* and tumorigenicity *in vivo*. These findings support our hypothesis that AFAP1-AS1 drives NPC tumorigenicity.

Our data also support the hypothesis that YAP stability is critical for AFAP1-AS1–driven cell proliferation in NPC. Previous studies have reported that AFAP1-AS1 promotes cell proliferation in non-small-cell lung cancer (31), colorectal cancer (32), triple-negative breast cancer (33), and esophageal squamous malignancies (34). However, the role and mechanism of AFAP1-AS1 in NPC pathogenesis remain poorly understood. AFAP1-AS1 has been reported to predict NPC survival prognosis and promote NPC metastasis through the inhibition of miR-423-5p (14). A study reported that AFAP1-AS1 depletion significantly suppresses NPC migration and invasion through the modification of the actin cytokeratin signaling pathway (15).

The Hippo-YAP signaling pathway is significantly altered after AFAP1-AS1 knockdown, as shown by the KEGG analysis performed using our RNA-Seq data. This links YAP signaling with NPC tumorigenesis, a finding consistent with those of previous reports (35). LncRNA THOR has also been reported to regulate YAP (36). This suggests that our AFAP1-AS1 model, which shows that lncRNA modulates YAP signaling, leading to enhanced NPC cell proliferation, may be feasible. Furthermore, YAP is reported to be a critical effector in the Hippo-YAP signaling pathway (37), and we observed that AFAP1-AS1 knockdown inhibited YAP expression. Additionally, lncRNA B4GALT1-AS1 enhanced YAP mRNA stability and promoted cell stemness and migration in osteosarcoma (38). We also found that AFAP1-AS1 stabilized YAP mRNA. In addition, RBM3, an RNA-binding protein, has been shown to function as an oncogene in many cancers (39, 40) and promote YAP mRNA stability (30). Using RNA-Seq analysis, we showed that RBM3 expression was downregulated due to KAT2B deletion. We also noted that AFAP1-AS1 stabilized YAP mRNA, which could be rescued by RBM3 overexpression. In conclusion, our results support the hypothesis that AFAP1-AS1 regulates NPC cellular proliferation through the stabilization of YAP mRNA.

Our study demonstrates that AFAP1-AS1 binds to KAT2B and enhances its acetyltransferase activity, which, in turn, upregulates TIF1α/H3K14ac complex formation and RBM3 transcription, thus leading to increased YAP mRNA stability. Previous studies have revealed that the KAT family protein KAT2A can bind to the lncRNAs GClnc1 and PVT1 in gastric cancer and NPC, respectively (6, 22). However, the specific KAT family protein member that binds to AFAP1-AS1 was unknown. Herein, we provide data suggesting that AFAP1-AS1 binds to KAT2B and validated these interactions using a KAT2B E570A/D610A mutant that suppressed AFAP1-AS1–enhanced KAT2B acetyltransferase activity. In accordance with previous reports on KAT2B histone acetylation modifications (30), our results revealed that KAT2B increased H3K14ac levels in NPC cells, while an F979A/N980A mutant in the bromodomain domain of TIF1α impaired TIF1α binding to H3K14ac, and inhibited AFAP1-AS1–induced and KAT2B-induced NPC cell proliferation. Moreover, the binding of TIF1α to H3K14ac was important for AFAP1-AS1–mediated YAP mRNA stabilization. We also determined that RBM3 expression stabilized YAP, and its overexpression rescued YAP stability that was inhibited by AFAP1-AS1 knockdown or KAT2B knockout. TIF1α and H3K14ac can directly bind to downstream gene promoters and enhance their expression (6, 30). We showed that TIF1α and H3K14ac directly promoted RBM3 transcription by binding to its promoter. These data help identify the mechanism of AFAP1-AS1–driven YAP mRNA stabilization as being mediated by KAT2B acetyltransferase activity and TIF1α/H3K14ac complex-promotion of RBM3 transcription inducing NPC cell proliferation

In conclusion, our data demonstrate that AFAP1-AS1 is a potential target for the development of novel therapeutic interventions in NPC. We uncovered a novel molecular mechanism wherein the KAT2B/H3K14ac/TIF1α/RBM3/YAP signaling pathway is important for AFAP1-AS1–driven NPC tumorigenicity. This information may contribute to the creation of personalized treatments for NPC patients.

## Data Availability Statement

The datasets presented in this study can be found in online repositories. The names of the repository/repositories and accession number(s) can be found in the article/**Supplementary Material**.

## Ethics Statement

The animal study was reviewed and approved by Institutional Ethical Review Board of the Zhengjiang Provincial People’s Hospital.

## Author Contributions

All authors were involved in the experiments for this paper. MF, JT, and YZ participated in the writing of the manuscript. All authors contributed to the article and approved the submitted version.

## Funding

This study was supported in part by grants from National Natural Science Foundation of China (Grant number: 81672430 to XM); National Natural Science Foundation of China (Grant number: 81960624 to YW); Zhejiang Provincial Nature Science Foundation of China (Grant number: LY18H160035 to XL); Zhejiang Provincial People’s Hospital Scientific Research Returned Foundation for The Excellent Youth (Grant number: ZRY2018B002 to JT); Zhejiang Provincial Nature Science Foundation of China (Grant number: LQ20H160063 to JT); Science and Technology Program of Gansu Province (Grant number: 18JR3RA262 to YW). Zhejiang Provincial Nature Science Foundation of China (Grant number: LY21H160051 to MF).

## Conflict of Interest

The authors declare that the research was conducted in the absence of any commercial or financial relationships that could be construed as a potential conflict of interest.
