# Supplementary material for: Long Noncoding RNA AFAP1-AS1 Is a Critical Regulator of Nasopharyngeal Carcinoma Tumorigenicity
Source: Front Oncol. 2020 Nov 23;10:601055. doi: 10.3389/fonc.2020.601055 (PMC7719841; doi:10.3389/fonc.2020.601055)
Supplement: Supplementary file 1 [file Image_1.pdf]

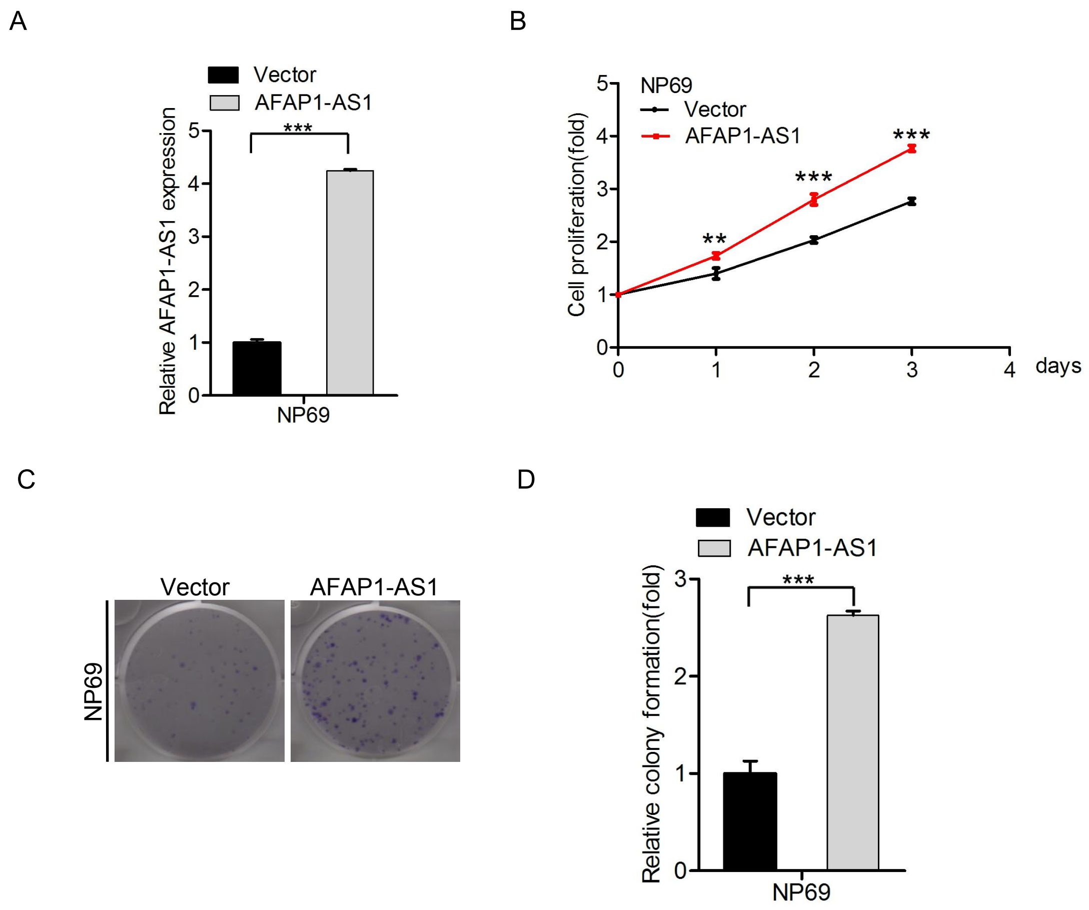

Supplementary Figure 1. **A**, qRT-PCR analysis of AFAP1-AS1 overexpression in NP69 cells. **B-C**, Representative results of cell proliferation (**B**) and colony formation assays (**C**) in NP69 cells transfected with AFAP1-AS1overexpressing plasmid or vector. **D**, Quantification of colony formation in (**C**). Error bars represent the SD. **P< 0.01. ***P< 0.001. Data are representative of three independent experiments.
