# Supplementary material for: Long Noncoding RNA AFAP1-AS1 Is a Critical Regulator of Nasopharyngeal Carcinoma Tumorigenicity
Source: Front Oncol. 2020 Nov 23;10:601055. doi: 10.3389/fonc.2020.601055 (PMC7719841; doi:10.3389/fonc.2020.601055)
Supplement: Supplementary file 2 [file Image_2.pdf]

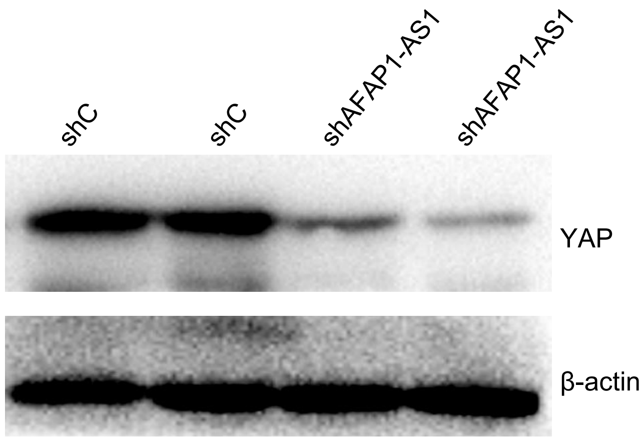

Supplementary Figure 2. WB analyses of effects of AFAP1-AS1 knockdown inhibited YAP expression in vivo. Tissues was from **Figure 2H**.
