# Supplementary material for: Long Noncoding RNA AFAP1-AS1 Is a Critical Regulator of Nasopharyngeal Carcinoma Tumorigenicity
Source: Front Oncol. 2020 Nov 23;10:601055. doi: 10.3389/fonc.2020.601055 (PMC7719841; doi:10.3389/fonc.2020.601055)
Supplement: Supplementary file 3 [file Image_3.pdf]

A

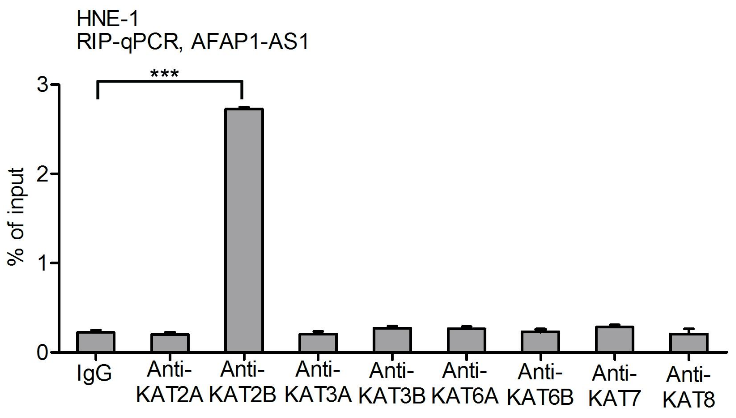

B

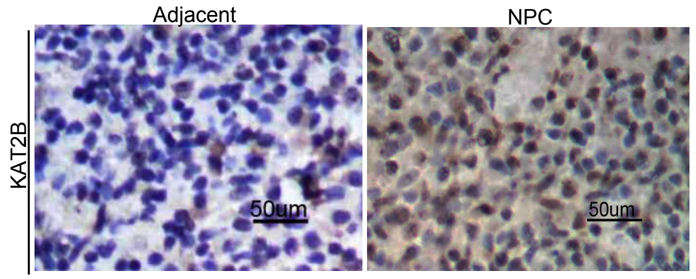

C

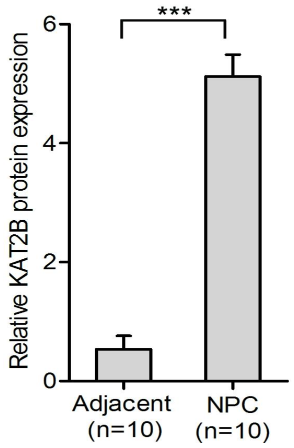

Supplementary Figure 3. A, Effects of the association of KAT2A, KAT2B, KAT3A, KAT3B, KAT6A, KAT7 or KAT8, and AFAP1-AS1. **B**, Immunohistochemistry staining assay of KAT2B in 10 pairs of freshly frozen NPC tumor and adjacent non-tumor specimens. **C**, Quantification analysis of KAT2B protein expression in (**B**). Error bars represent the SD. ***P< 0.001. Data are representative of three independent experiments.
