# Supplementary material for: Long Noncoding RNA AFAP1-AS1 Is a Critical Regulator of Nasopharyngeal Carcinoma Tumorigenicity
Source: Front Oncol. 2020 Nov 23;10:601055. doi: 10.3389/fonc.2020.601055 (PMC7719841; doi:10.3389/fonc.2020.601055)
Supplement: Supplementary file 4 [file Image_4.pdf]

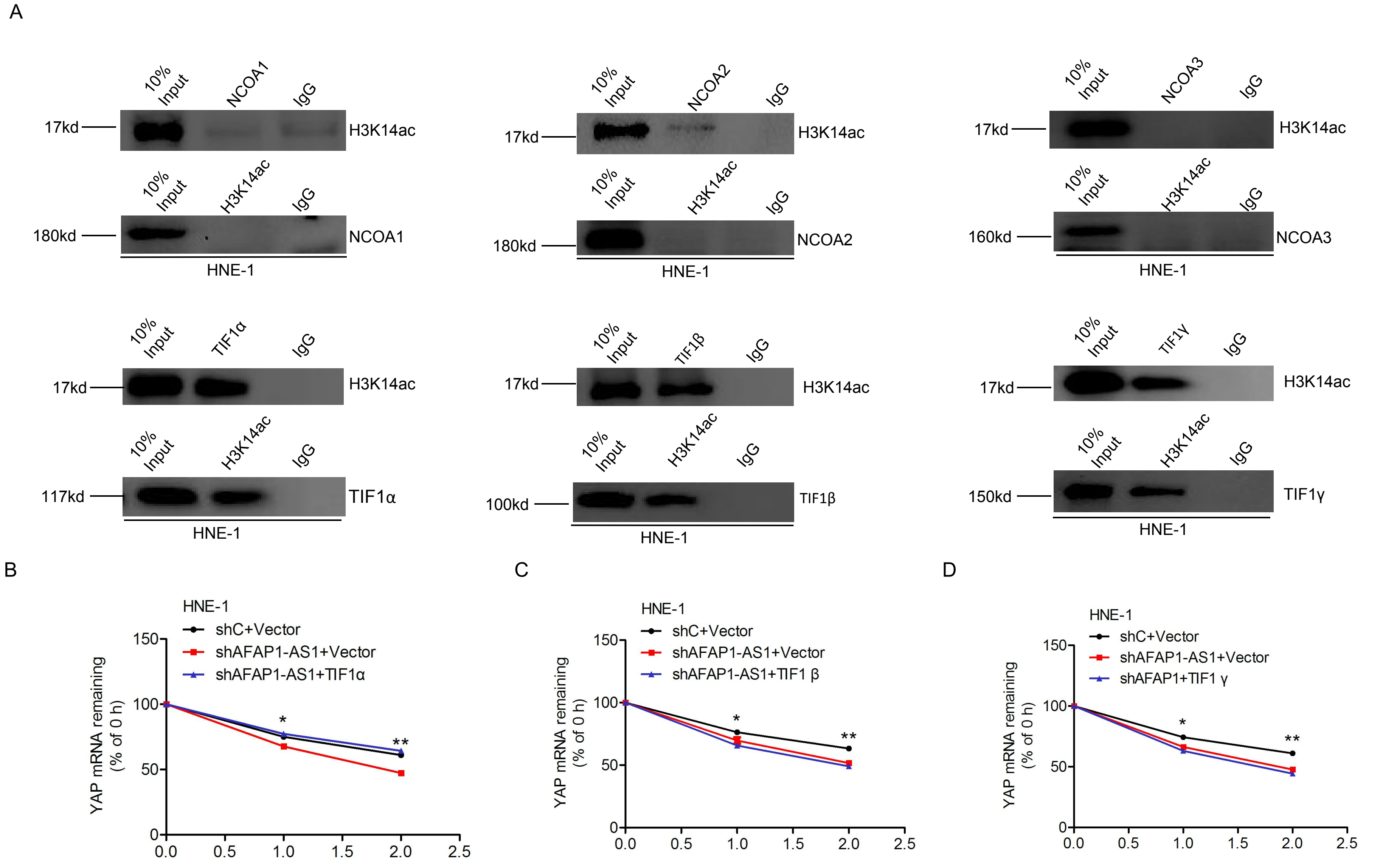

Supplementary Figure 4. A, Immunoprecipitation and WB analyses of effects of H3K14ac association with NCOA1, NCOA2, NCOA3, TIF1α, TIF1β, and TIF1γ. **B-D**, TIF1α but not TIF1β and TIF1γ rescues AFAP1-AS1 knockdown-inhibited YAP mRNA stability. Error bars represent the SD. *P<0.05. **P<0.01. Data are representative of three independent experiments.
