# Supplementary material for: Long Noncoding RNA AFAP1-AS1 Is a Critical Regulator of Nasopharyngeal Carcinoma Tumorigenicity
Source: Front Oncol. 2020 Nov 23;10:601055. doi: 10.3389/fonc.2020.601055 (PMC7719841; doi:10.3389/fonc.2020.601055)
Supplement: Supplementary file 5 [file Image_5.pdf]

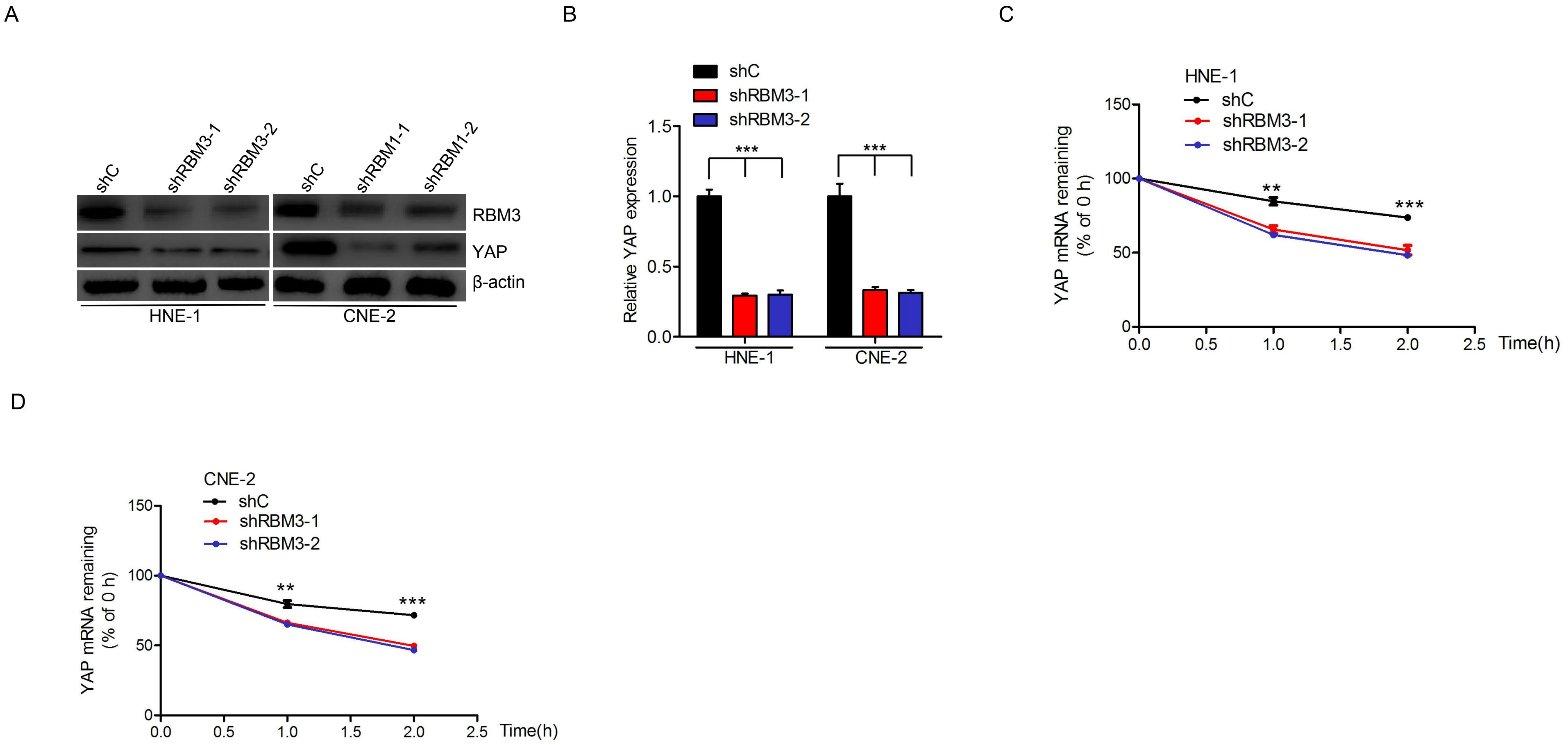

Supplementary Figure 5. A-D, Effects of RBM3 knockdown on YAP protein and mRNA expression, mRNA stability in HNE-1 and CNE-2 cells. Error bars represent the SD. **P<0.01. ***P<0.001. Data are representative of three independent experiments.
