# Supplementary material for: Long Noncoding RNA AFAP1-AS1 Is a Critical Regulator of Nasopharyngeal Carcinoma Tumorigenicity
Source: Front Oncol. 2020 Nov 23;10:601055. doi: 10.3389/fonc.2020.601055 (PMC7719841; doi:10.3389/fonc.2020.601055)
Supplement: Supplementary file 6 [file Table_1.docx]

**Supplementary Table 1** Primers for qRT-PCR assays and ChIP-qPCR assays

| Primer pairs | Sequence |
| --- | --- |
| qRT-PCR,β-actin | 5’-CATGTACGTTGCTATCCAGGC-3’ and  5’-CTCCTTAATGTCACGCACGAT-3’ |
| qRT-PCR,YAP | 5’-TAGCCCTGCGTAGCCAGTTA-3’ and  5’- TCATGCTTAGTCCACTGTCTGT-3’ |
| qRT-PCR,RBM3 | 5’-GAGGGCTCAACTTTAACACCG-3’ and  5’-GACCACCTCAGAGATAGGTCC-3’ |
| qRT-PCR,AFAP1-AS1 primer 1 | 5’-GCATCAAATCATGACCTGG-3’ and  5’-GTTCCTGACATGCTTTCTC-3’ |
| qRT-PCR,AFAP1-AS1 primer 2 | 5’-CGTTCACTTCAATAGCCGC-3’ and  5’-GGAGAAGGGATCGTCCCAT-3’ |
| ChIP-qPCR,  RBM3 promoter  -1918 to -1702 | 5’-AGGCGGAGGTTGCAGTG-3’ and  5’-GTCTAATTATTAATCCC-3’ |
| ChIP-qPCR,  RBM3 promoter  -1567 to -1343 | 5’-TTAGCCTCTGAGGGCAA-3’ and  5’-AAGACTCAGTCTCAAA-3’ |
| ChIP-qPCR,  RBM3 promoter  -1325 to -1087 | 5’-GGAGTGCAGTGGCATGAT-3’ and  5’-CCTGTAATCCCAGCACTT-3’ |
| ChIP-qPCR,  RBM3 promoter  -879 to -643 | 5’-AGTAGGTGGGACTACAG-3’ and  5’-ACTGCCTGAGATGGCTT-3’ |
| ChIP-qPCR,  RBM3 promoter  -412 to -176 | 5’-TCAGGCCTTTAAAGGGTATG-3’ and  5’-GGTGCACCCTAAACGTCAGC-3’ |
