# Supplementary material for: Long Noncoding RNA AFAP1-AS1 Is a Critical Regulator of Nasopharyngeal Carcinoma Tumorigenicity
Source: Front Oncol. 2020 Nov 23;10:601055. doi: 10.3389/fonc.2020.601055 (PMC7719841; doi:10.3389/fonc.2020.601055)
Supplement: Supplementary file 7 [file Table_2.docx]

**Supplementary Table 2** Mass spectrometry analysis of the proteins pulled down by biotin-AFAP1-AS1 in HEK-293T cells

| Number | Protein name | Cover Percent Diff (MH+) |
| --- | --- | --- |
| 1 | Vimentin | 74.5% |
| 2 | KAT2B | 67.8% |
| 3 | ANXA2 | 67.3% |
| 4 | PFN1 | 65.7% |
| 5 | TUBA1B | 57.9% |
| 6 | RPL12 | 54.5% |
| 7 | KRT9 | 50.7% |
| 8 | EIF5A | 50% |
| 9 | RPS12 | 58.5% |
| 10 | KRT14 | 47.2% |
